# T cell-specific constitutive active SHP2 enhances T cell memory formation and reduces T cell activation

**DOI:** 10.3389/fimmu.2022.958616

**Published:** 2022-08-02

**Authors:** Clemens Cammann, Nicole Israel, Sarah Frentzel, Andreas Jeron, Eylin Topfstedt, Thomas Schüler, Luca Simeoni, Martin Zenker, Hans Joerg Fehling, Burkhart Schraven, Dunja Bruder, Ulrike Seifert

**Affiliations:** ^1^ Friedrich Loeffler-Institute for Medical Microbiology-Virology, University Medicine Greifswald, Greifswald, Germany; ^2^ Institute of Molecular and Clinical Immunology, Health Campus Immunology, Infectiology and Inflammation, Otto-von-Guericke-University Magdeburg, Magdeburg, Germany; ^3^ Institute of Medical Microbiology, Infection Prevention and Control, Infection Immunology Group, Health Campus Immunology, Infectiology and Inflammation, Ottovon-Guericke-University Magdeburg, Magdeburg, Germany; ^4^ Immune Regulation Group, Helmholtz Center for Infection Research, Braunschweig, Germany; ^5^ Institute of Human Genetics, Otto-von-Guericke University Magdeburg, Magdeburg, Germany; ^6^ Institute of Immunology, University Hospital Ulm, Ulm, Germany

**Keywords:** T cell receptor signaling, Src homology region 2 domain-containing protein-tyrosine phosphatase 2 (SHP2), SHP2 gain-of-function mutation, T lymphocyte, effector memory T cells (TEMs)

## Abstract

Upon antigen recognition by the T cell receptor (TCR), a complex signaling network orchestrated by protein-tyrosine kinases (PTKs) and protein-tyrosine phosphatases (PTPs) regulates the transmission of the extracellular signal to the nucleus. The role of the PTPs Src-homology 2 (SH2) domain-containing phosphatase 1 (SHP1, *Ptpn6*) and Src-homology 2 (SH2) domain-containing phosphatase 2 (SHP2, *Ptpn11*) have been studied in various cell types including T cells. Whereas SHP1 acts as an essential negative regulator of the proximal steps in T cell signalling, the role of SHP2 in T cell activation is still a matter of debate. Here, we analyzed the role of the constitutively active SHP2-D61Y-mutant in T cell activation using knock-in mice expressing the mutant form *Ptpn11^D61Y^* in T cells. We observed reduced numbers of CD8^+^ and increased numbers of CD4^+^ T cells in the bone marrow and spleen of young and aged SHP2-D61Y-mutant mice as well as in Influenza A Virus (IAV)-infected mice compared to controls. In addition, we found elevated frequencies of effector memory CD8^+^ T cells and an upregulation of the programmed cell death protein 1 (PD-1)-receptor on both CD4^+^ and CD8^+^ T cells. Functional analysis of SHP2-D61Y-mutated T cells revealed an induction of late apoptosis/necrosis, a reduced proliferation and altered signaling upon TCR stimulation. However, the ability of D61Y-mutant mice to clear viral infection was not affected. In conclusion, our data indicate an important regulatory role of SHP2 in T cell function, where the effect is determined by the kinetics of SHP2 phosphatase activity and differs in the presence of the permanently active and the temporally regulated phosphatase. Due to interaction of SHP2 with the PD-1-receptor targeting the protein-tyrosine phosphatase might be a valuable tool to enhance T cell activities in immunotherapy.

## Introduction

The Src homology 2 domain-containing phosphatase 2 (SHP2) belongs to the non-receptor protein-tyrosine phosphatase family and exerts important functions in cell survival, proliferation, migration, and differentiation in many tissues. Mutations within the *Ptpn11* gene locus encoding SHP2 promote tumor progression and have been associated with Noonan syndrome, juvenile myelomonocytic leukemia (JMML) (1, 2) and various other cancers (3, 4). Enhanced activation of the Ras/Erk signaling pathway downstream of several growth factor receptors appears to be responsible for tumorigenesis induced by *Ptpn11* gain-of-function mutations (5). This is accompanied by increased cell division and DNA damage in SHP2 mutant cells (6). Based on these observations SHP2 is a potential therapeutic target in cancer therapy and mutated SHP2 plays a significant role in promoting chemoresistance (7, 8). On the other side, SHP2 can act as a tumor suppressor as observed in hepatocellular carcinogenesis (9). In hematopoietic stem cells *Ptpn11 -* deficiency causes apoptosis which results in bone marrow aplasia (10).

In T cells SHP2 has been hypothesized to fine-tune T cell signaling processes by modulating the activation threshold through binding to programmed cell death 1 (PD-1) receptor but also by sustaining downstream Erk1/2 MAPK activity (11, 12). On the other side, protein-tyrosine phosphatase SHP1 plays a crucial role in downregulating the T cell signaling cascade by de-phosphorylation of proximal signaling proteins (13–16). Interaction of SHP2 with PD-1 results in diminished T cell function as observed by reduced interleukin (IL)-2 production (17–20). A possible explanation for these findings is that SHP2 is sequestered by PD-1 with the consequence that SHP2 is no longer able to remove inhibitory phosphate groups from signaling proteins such as lymphocyte-specific protein tyrosine kinase Lck and downstream proteins involved in the Ras/Erk cascade (18, 21). Analyses of mutant mice expressing dominant-negative SHP2 in T cells indicated an important function of SHP2 in skewing T cell differentiation towards the Th2 subset (22). In contrast, recently published data indicate that SHP2 is dispensable for establishing T cell exhaustion and the formation of virus-specific CD8^+^ T cells (23, 24). These divergent results may be due to different mouse models used in the above-mentioned studies. Indeed, Nguyen et al. (12) used conditional knock-out mice in which SHP2 was deleted by crossing SHP2 to Lck-Cre transgenic mice, whereas Rota et al. (23) and Miah et al. (24) employed CD4-Cre transgenic mice in most of their experiments to delete SHP2.

In several studies activating mutations of SHP2 such as D61Y, D61G or E76K were investigated. Mice carrying the E76K mutation in all hematopoietic cells developed a myeloproliferative disorder (MPD) and displayed effects of malignant transformation in several hematopoietic lineages with the initiation of acute leukemia in myeloid cells as well as in T and B cells (25). Similar observations were made by analyzing murine models with the germline gain-of-function mutation D61G and a conditional knock in of D61Y in hematopoietic cells, which emerged with myeloproliferative disorders comparable to that observed in JMML patients (26, 27).

For a T cell specific analysis of the SHP2-D61Y-mutation we used the pTα-Cre mice, where the Cre recombinase is expressed under the *Ptcra* locus in pTα^+^ thymocytes (28). The D61Y gain-of-function mutation leads to a constant open conformation of SHP2 where the two SH2 domains are accessible to protein binding and the phosphatase domain remains constantly active. This could be further verified in a computational study in which the molecular dynamics of the D61Y mutation on the SHP2 protein was simulated (29). Although expression of the leukemia-associated mutant *Ptpn11^D61Y^* in T cells did not result in myeloproliferative disorder *in vivo*, we observed an increased number of effector memory CD8^+^ T cells and an enhanced PD-1 receptor expression on CD4^+^ and CD8^+^ T cells. In addition, T cells bearing the SHP2-D61Y-mutant exhibited reduced T cell activation and proliferation. Together, our data support the observation that SHP2 plays an important role in defining the threshold of T cell activation, with the level of SHP2 activity being a critical factor.

## Methods

### Animals and influenza a virus infection

Conditional SHP2^D61Yflox^ (B6.129S6-Ptpn11tm1Toa/Mmjax) mice were kindly provided by B. G. Neel and T. Araki (30). Mice were bred on C57BL/6J background and crossed to CD4-Cre (B6.Cg-Tg(CD4-cre)1Cwi/BfluJ, Jackson Laboratory) or Ptcra^tm1(icre)Hjf^ generated by Luche et al. (28). Both strains were bred in the Central Animal Facility of Magdeburg and the Central Animal Facility Institution of the University of Greifswald. Mice were treated in accordance with the German National Guidelines for the Use of Experimental Animals. For genotyping the SHP2^D61Y^ mutation was detected by a melting point analysis using the High Resolution Melting and Gene scanning application on the LightCycler 480 (Roche Diagnostics) as described before (**Supplementary Figure 1A**) (31). Protein expression of SHP2 was comparable between pTα^SHP2mut^ and pTα^SHP2wt^ mice (**Supplementary Figure 1B**).

Unless otherwise stated, mice aged 10 to 14 weeks were used for steady state analysis, comparing SHP2^D61Yflox/wt^-pTa-Cre (hereinafter referred to as pTα^SHP2mut^ mice) and SHP2^D61Ywt/wt^-pTa-Cre mice (referred to as pTα^SHP2wt^) as control. For Influenza A Virus (IAV) infection, the PR8-OT-I strain (A/Puerto Rico/8/34 virus, PR8; H1N1) containing the SIINFEKL-epitope (OVA_257-264_) was used (32). Eight- to twelve-week-old pTα^SHP2mut^ and pTα^SHP2wt^ mice were anaesthetized *via* intraperitoneal injection of ketamine/xylazine followed by intranasal infection of mice using 2.5 foci forming units of the PR8-OT-I virus suspended in 25 µl sterile PBS.

### Cell isolation

Mice were deeply anesthetized with Isoflurane and perfused transcardially using 5 mL ice cold Dulbecco’s Phosphate-Buffered Saline (DPBS, Gibco). Spleen, BM, thymus, lungs and lymph nodes were removed quickly and collected in 3 mL DPBS supplemented with 2% FCS on ice. Afterwards, thymocytes, splenocytes, cells of lymph nodes and BM cells were isolated non-enzymatically. Therefore, organs were mechanically homogenized through a 40 µm cell strainer and femuri were flushed using a sterile 27Gx^3/4^ needle (BD) and 5 mL syringe. To lyse erythrocytes, splenocytes were treated for 1 ½ - 2 min with 2 mL of Ammonium-chloride-potassium buffer on ice. Lung infiltrating leucocytes were isolated enzymatically. To isolate leucocytes from lungs, tissue was minced and incubated for 30 min at 37°C using Iscove’s modified Dulbecco’s medium (Life Technologies) supplemented with 0.2 mg/mL Collagenase D (Roche) and 0.01 mg/mL DNase I (Sigma-Aldrich). Digestion was stopped using 5 mM EDTA. Afterwards, cells were filtered using a 70 µm cell strainer, centrifuged and resuspended in 35% Easycoll (Density: 1.124 g/m, Biochrom). Cells were then centrifuged for 6 min at 300 x g without brakes and at room temperature. Living cells were counted using a Neubauer chamber and trypan blue staining and subsequently adjusted for further analysis.

For cell death analysis, proliferation assays and western blot analysis of signaling proteins T cells from spleens were magnetically sorted by negative selection using the pan T cell Isolation Kit (Miltenyi Biotech) following manufacturer’s instruction and purified using AutoMACSpro (Miltenyi Biotec).

### Cell death/apoptosis assay

Following purification, 5x10^5^ cells were incubated in the presence of 5 µg/mL plate-bound α-CD3 (clone 145-2C11, BioLegend) and 1µg/mL soluble α-CD28 mAB (BioLegend) for 24 and 48 hours at 37°C. Cell death was determined using AnnexinV and PI staining Kit (BioLegend) according to manufacturer’s instruction. Cells were acquired immediately using a BD LSRIIFortessa flow cytometer and Data were analyzed *via* FlowJo (v10.4 Treestar).

### Proliferation assay

To assess the proliferative capacity, cells were cultivated in 96-well round-bottomed tissue culture plates coated with 1 µg/mL or 5 µg/mL anti-CD3 for 72 hours. ^[3H]^Thymidine (0.3 μCi/well; specific activity, 50 Ci/mmol) was added for the last 8 to 10 hours, and the plates were harvested using a PHD cell harvester (Inotech AG, Basel, Switzerland). Thymidine incorporation was measured by liquid scintillation counting.

### Western blot

Following purification, 2x10^6^ cells were incubated with biotinylated anti-CD3 antibody (clone 145-2C11, BioLegend) and cross-linked with streptavidin (BioLegend) for 3, 10 and 60 minutes. Samples were lysed in buffer containing 1% lauryl maltoside (N-dodecyl β-maltoside), 1% NP-40, 1 mM Na_3_VO_4_, 1 mM PMSF, 10 mM NaF, 10 mM EDTA, 50 mM Tris pH 7.5, and 150 mM NaCl. Proteins were separated using SDS-Page and transferred to a nitrocellulose membrane (GE Healthcare). Membranes were incubated overnight with the following primary antibodies ERK, pERK (Thr202/Tyr204), JNK, pJNK (Thr183/Tyr185), pPLCγ (Y783), ZAP70, pZAP70 (Y319), LAT, pLAT (Y191) and GAPDH (all Cell Signaling Technology). Afterwards, membranes were incubated with corresponding horseradish peroxidase-conjugated secondary antibodies α-mouse (115-035-044, Dianova) or α-rabbit (111-035-003, Dianova) for 1 h and subsequently visualized by enhanced chemiluminescence (Cell Signaling Technology) using FusionFX7 technology (peqLab).

### Flow cytometry

Prior to immunostaining, isolated leukocytes were incubated with 0.5 µg/mL anti-FcγIII/II receptor antibody and Zombie Nir™ Fixable Viability Dye (BioLegend) for 20 minutes. Afterwards cells were washed and stained with indicated surface antibodies obtained from BioLegend for 30 minutes in the dark: CD3 (clone 145-2C11), CD4 (clone GK1.5 or RM4-5), CD8 (clone 53-6.7), CD25 (clone 3C7), CD44 (clone IM7), CD62L (clone MEL-14). To analyze IAV-specific T cells, cells were incubated with fluorescence-coupled H-2Kb SIINFEKL MHC Pentamer (Ovalbumin_257-264_, Proimmune) according to manufacturer instruction. For intracellular cytokine detection, cells were incubated for 16 hours with 5µg/mL plate-bound anti-CD3 and 1µg/mL soluble CD28. For IFNγ (clone XMG1.2, BioLegend) staining, eBioscience Foxp3/Transcription Factor Staining Buffer Set (Thermofisher) was used according to manufacturer instruction. All steps were performed on ice. Samples were acquired using a BD LSRIIFortessa flow cytometer and data were analyzed *via* FlowJo (v10.4 Treestar).

### qRT-PCR of viral load

Following isolation, lungs were mechanically minced and tissue was stored in Allprotect Tissue Reagent (Qiagen). Tissue was transferred to RLT lysis buffer (RNeasy, Qiagen) and homogenized. RNA was isolated according to manufacturer's instruction using RNeasy^®^ Mini Kit including DNase I digestion (Qiagen). cDNA was generated using Maxima First Strand cDNA Synthesis Kit for RT-qPCR (Thermofisher) according to manufacturer's instruction. Absolute viral load was quantified using SYBR Green approach (FastStart Essential DNA Green Master, Roche) and the following primers for IAV nucleoprotein (forward 5’-GAGGGGTGAGAATGGACGAAAAAC-3’ and reverse 5’-CAGGCAGGCAGGCAGGACTT-3’). All samples were measured in triplicates using LightCycler^®^ 480 Instrument II (Roche) and a standard cDNA dilution series was tested in duplicates for each qPCR experiment performed.

### Statistical analysis

Graphs were generated by using GraphPad Prism software version 7. Data are presented as mean + SD. Statistical significance was calculated using the unpaired two-tailed Student’s t-test. Values of p < 0.05; 0.01; 0.001 or 0.0001 were marked by one, two, three or four asterisks, respectively.

## Results

### SHP2 gain-of-function mutation D61Y in T cells does not cause JMML-like symptoms

**Figure 1:**
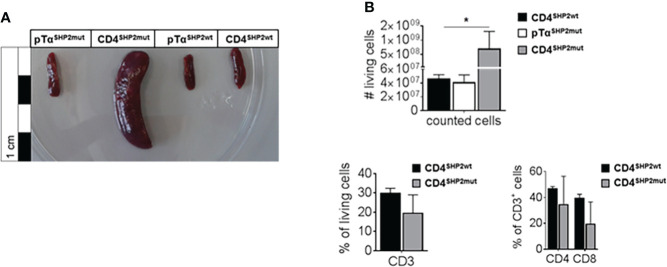
SHP2 mutation induces splenomegaly and increased cell numbers in CD4-Cre but not in pTα-Cre mice. CD4^SHP2mut^, pTα^SHP2mut^ and Cre-control mice were kept for 11 to 14 months; spleens were removed, photographed and further analyzed by flow cytometry. **(A)** Splenomegaly was observed in CD4^SHP2mut^ mice. **(B)** Splenocytes of aged naïve mice were isolated, gated on single living cells and used for further FACS analysis. Bar graph shows total cell numbers of pTα^SHP2mut^ mice, CD4^SHP2mut^ or Cre-control mice (upper panel) and frequencies of CD3^+^ T cells as well as frequencies of CD4^+^ and CD8^+^ T cells within the CD3^+^ T cell population for CD4^SHP2mut^ compared to control mice (lower panels). Data are representative for 2 independent experiments with n = 2 SHP2 mutant or 3 control mice. (*p<0.05, Student’s t-test, mean + SD).

In mice, expression of the SHP2-D61Y mutation in the hematopoietic compartment results in severe myeloproliferative disease, disruption of splenic architecture and cell infiltration into organs such as the liver. Therefore, the D61Y mutation serves as a potential model to mimic the human condition of JMML (26). Similar to those studies, 1-year old CD4^SHP2mut^ mice developed severe splenomegaly (**Figure 1A**). Analyses of the spleens of control and CD4^SHP2mut^ mice revealed increased cell numbers but rather comparable frequencies of T cells and T cell subsets (**Figure 1B**). Since CD4 is not exclusively expressed in T cells but also in a subset of macrophages and DCs (33–35), the question remained whether the observed expansion is T cell-specific or an indirect effect of hyper-proliferating myeloid cells. To exclude effects raised by macrophages and other antigen presenting cells, we further crossed the conditional SHP2^D61Y^floxed mice to pre-T cell receptor α (pTα) mice. In these mice the receptor is expressed only in early thymic development of T cells thus ensuring that the SHP2-D61Y mutation is exclusively evident in these cells. In contrast to mice crossed to CD4-Cre animals, pTα^SHP2mut^ mice did not develop splenomegaly and cell numbers were comparable to those isolated from control mice indicating that the development of JMML-like features is rather the result of the gain-of-function mutation in myeloid cells than the result of a T cell intrinsic mechanism (**Figures 1A, B**).

Previous studies of Lck-dependent deletion of SHP2 in T cells revealed an impaired transition from the double-negative (DN) stage DN3 to DN4 during thymic T cell development, leading to significantly reduced frequencies of CD4^+^/CD8^+^ double-positive (DP) cells (12). This observation pointed to a role of SHP2-mediated signaling during thymic maturation. Analyses of T cell development in thymi obtained from CD4^SHP2mut^ mice expressing the gain-of-function mutation D61Y and CD4^SHP2wt^ control mice revealed no alterations in CD4^+^/CD8^+^ double- or single-positive cells and in the general amount of double-negative T cells but demonstrated a reduction in T cells reaching the DN4 stage of development where CD4 is first expressed (data not shown). To clarify whether this observation is caused by SHP2 gain-of-function mutation in T cells or due to secondary effects of other CD4^+^ cells such as DCs carrying the D61Y mutation, thymi of pTα^SHP2mut^ were analyzed. As observed for CD4^SHP2mut^ mice, also pTα^SHP2mut^ revealed no alterations in CD4^+^/CD8^+^ double-positive and single-positive cells as well as in the amount of double-negative T cells. In contrast to the data obtained in CD4^SHP2mut^ mice, analysis of the different DN stages, characterized by the expression of CD44 and CD25, also showed no significant differences between both pTα^SHP2mut^ and Cre-control (pTα^SHP2wt^) mice (**Supplementary Figure 2**). In conclusion the D61Y mutation in CD4-Cre mice led to significant phenotypic differences characterized by increased total cell numbers resulting in splenomegaly already under steady state conditions, which cannot be observed in T cell specific pTα-Cre mice carrying the same mutation. To focus on the role of SHP2 in T cells, we therefore performed all further experiments using the pTα^SHP2mut^ mice.

### T cell-specific expression of SHP2 mutation D61Y increases T cell CD44^int^ population and memory formation

**Figure 2:**
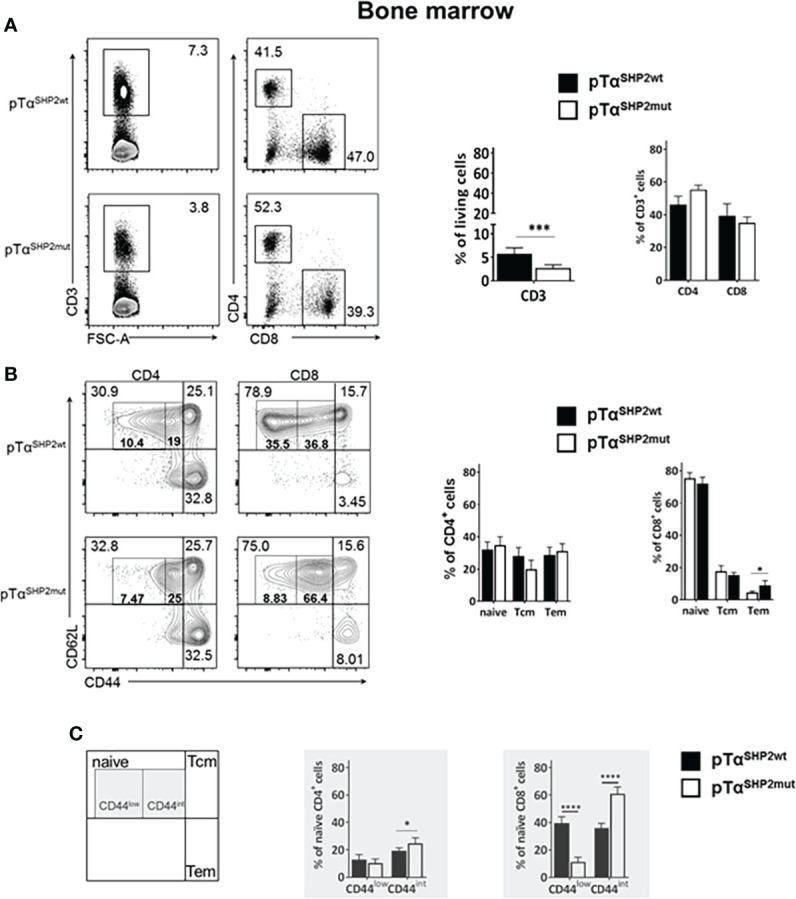
Alterations in T cell composition within the bone marrow of SHP2-D61Y mutant mice. Cells isolated from bone marrow of naïve pTα^SHP2mut^ and Cre-control mice were gated on single living cells and used for further FACS analysis. **(A)** FACS plots and graphs show frequencies of CD3^+^ T cells within the bone marrow and the CD4:CD8 T cell ratio in pTα^SHP2wt^ and pTα^SHP2mut^ mice. **(B)** FACS plots and graphs show proportion of naïve, central memory and effector memory T cells in both CD4^+^ and CD8^+^ T cell subsets. **(C)** CD44^low^ expression in naïve T cells and CD44 intermediate expression in both CD4^+^ and CD8^+^ T cells in the bone marrow, marked by grey squares. Data is representative for 2 independent experiments with n=5 mice each. (*p<0.05, ***p<0.001, ****p<0.0001, Student’s t-test, mean + SD).

**Figure 3:**
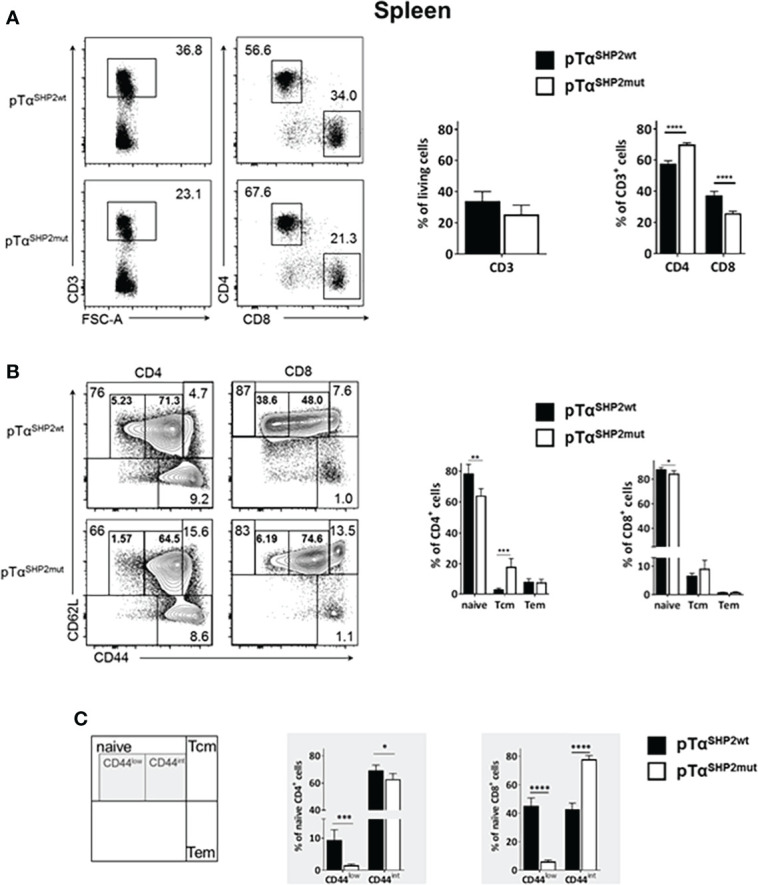
Alterations in T cell composition within the spleen of SHP2-D61Y mutant mice. Cells isolated from spleen of naïve pTα^SHP2mut^ and pTα^SHP2wt^ control mice were gated on single living cells and used for further FACS analysis. **(A)** FACS plots and graphs show frequencies of CD3^+^ T cells within the spleen and a shift in the CD4:CD8 T cell ratio. **(B)** FACS plots and graphs show proportion of naïve, central memory (Tcm) and effector memory T cells (Tem) in both CD4^+^ and CD8^+^ T cell subsets. **(C)** Frequencies of CD44^low^ and CD44 intermediate (CD44^int^) naïve T cells in the spleen, marked by grey squares. Data is representative for 2 independent experiments with n=5 mice each. (*p<0.05, **p<0.01, ***p<0.001, ****p<0.0001, Student’s t-test, mean + SD).

Although thymic T cell development was not altered upon pTα^SHP2mut^ gain-of-function mutation, significantly lower frequencies of CD3^+^ T cells were found in the bone marrow of pTα^SHP2mut^ mice compared to pTα^SHP2wt^ mice (**Figure 2A**). In the spleen, only a tendency in CD3^+^ T cell reduction was visible in pTα^SHP2mut^ mice, whereas significant lower frequencies of splenic CD8^+^ T cells and increased CD4^+^ T cell level were detected compared to the spleens of pTα^SHP2wt^ mice (**Figure 3A**). Similar results were obtained for the analyzed lymph nodes (**Supplementary Figure 3A**). Examination of naïve and memory T cell subsets, characterized by the expression of the adhesion markers CD62L and CD44, demonstrated a shift from naïve to central memory T cell (Tcm) phenotype in CD4^+^ T cells in the spleen, whereas CD8^+^ T cells revealed a shift in the ratio of naïve T cells to effector memory T cells (Tem) in the bone marrow (**Figures 2B**, **3B**). More strikingly, in both organs, the abundance of CD4^+^ and CD8^+^ T cells expressing elevated levels of CD44 was increased in pTα^SHP2mut^ mice, which was accompanied by a reduction of the CD44^low^ T cell population (except for naïve CD4^+^ CD44^int^ T cells in the spleen). In summary, these data indicate that either memory T cell formation is induced in the presence of the D61Y mutation, or a change in the activation threshold leads to an altered homeostatic proliferation and increased apoptosis of naïve CD44^low^ T cells under non-infectious steady state conditions (**Figures 2C**, **3C**).

During aging, higher numbers of memory T cells are detectable. Whereas in early life the majority of T cells is naïve and newly emerged from the thymus, memory T cells are generated upon exposure to antigen and begin to accumulate during early life with a plateau in adulthood. To analyze whether alterations within the T cell population can be found in aged mice bearing the SHP2 gain-of-function mutation, pTα^SHP2mut^ and pTα^SHP2wt^ control mice were maintained for 11-14 months. In both, spleen and bone marrow, a significant reduction of CD3^+^ T cells and a significant shift in the CD4:CD8 T cell ratio with lower frequencies of CD8^+^ T cells and elevated CD4^+^ T cells was observed in pTα^SHP2mut^ mice compared to Cre-controls (**Supplementary Figures 3B, C**). Whereas frequencies of naïve and memory T cells within the CD4^+^ compartment of pTα^SHP2mut^ mice remained unchanged, CD8^+^ T cells showed a pronounced shift from lower frequencies of naïve to higher frequencies of Tem cells in both, spleen and bone marrow (**Supplementary Figures 3D, E**), and a significant increase in Tcm cells within the spleen (**Supplementary Figure 3D**). Notably, enhanced numbers of CD4^+^ and CD8^+^ T cells expressing the inhibitory immune checkpoint receptor PD-1 could be observed in spleens but not in the bone marrow of pTα^SHP2mut^ mice (**Supplementary Figures 3F, G**).

### Enhanced apoptosis and altered signaling upon TCR engagement in T cells carrying the D61Y-mutation

As shown in representative flow cytometry plots of the bone marrow of 10-14 weeks-old pTα^SHP2mut^ mice (**Figure 2A**) and of the spleen and the bone marrow of 11-14 months-old pTα^SHP2mut^ mice (**Supplementary Figures 3B, C**), CD3^+^ T cell frequencies are reduced indicating an altered homeostasis in the T cell compartment of pTα^SHP2mut^ mice.

**Figure 4:**
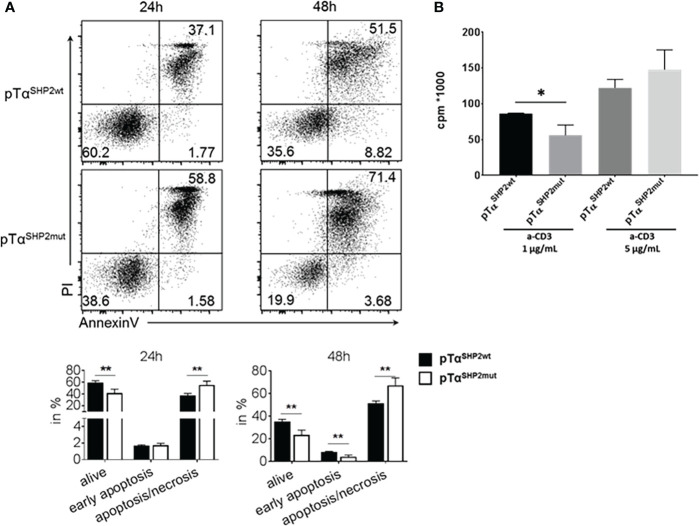
Increased late apoptosis/necrosis and reduced cell proliferation upon TCR triggering in pTα^SHP2mut^ mice. T cells were purified from splenocytes and incubated for the indicated time points. **(A)** T cells were incubated for 24 or 48 h with plate-bound α-CD3 and soluble CD28 and stained with AnnexinV and PI. FACS plots and graphs show increased late apoptosis (AnnexinV, PI double positive staining) in T cells with the SHP2-D61Y mutation. Data are representative for 3 independent experiments with n=3 control and 3 pTα^SHP2mut^ mice. **(B)** T cells were incubated with 1 or 5µg/mL α-CD3 for 72 h, proliferation was assessed by 3^H^thymidine incorporation. Data are representative for 2 independent experiments with n=3 mice each. (*p<0.05, **p<0.01, Student’s t-test, mean + SD).

**Figure 5:**
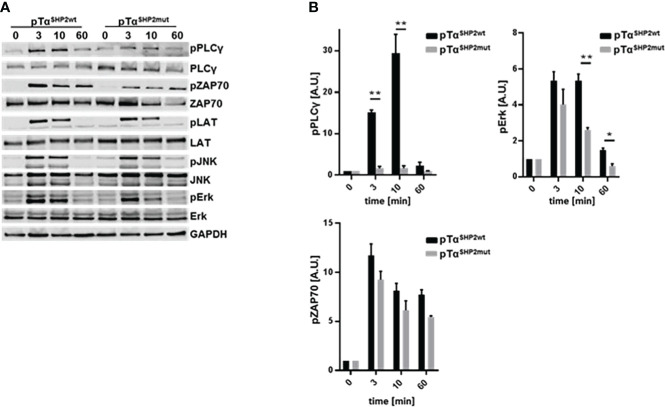
Reduced phosphorylation of PLCγ and Erk upon TCR triggering in pTα^SHP2mut^ mice. **(A)** Pan T cells were purified from splenocytes and components of the TCR signaling cascade were analyzed after 0, 3, 10 and 60 minutes following soluble α-CD3 (5µg/ml) stimulation *via* immunoblot, n=3. One representative experiment out of three is shown. **(B)** Densitometric analysis of the phosphorylation of PLCγ, ZAP70 and Erk, band intensities were normalized to the corresponding non-phosphorylated protein and the loading control GAPDH. (*p<0.05, **p<0.01, Student’s t-test, mean + SD).

To assess potential mechanisms for increased T cell turnover in pTα^SHP2mut^ mice activation-induced cell death was determined. T cells obtained from the spleens of pTα^SHP2mut^, and Cre-control mice were purified and treated with 5 µg/mL plate-bound anti-CD3 and 1µg/mL soluble CD28 antibodies. Annexin V and PI staining confirmed that more T cells obtained from pTα^SHP2mut^ mice underwent late-stage apoptosis/necrosis after 24h and 48h of stimulation than T cells isolated from Cre-control mice (**Figure 4A**). In addition, 72h cultivation of pTα^SHP2mut^ T cells with 1 µg/mL plate-bound anti-CD3 antibody revealed a significant reduction in the proliferative capacity of T cells with the D61Y mutation. However, this effect was only detectable at a low anti-CD3 (clone 145-2C11) concentration, whereas higher concentrations such as 5 µg/mL or the presence of costimulatory CD28 showed no differences between pTα^SHP2mut^ and Cre-control T cells (**Figure 4B**). These observations indicate that there might be an increased threshold to activate the SHP2-D61Y mutation-bearing T cells. This could prevent the induction of signaling events upon low signal peptide/MHC-TCR interaction which was shown to be required for homeostatic proliferation of naïve T cells (36). To further investigate this function, we analyzed the impact of the constantly active SHP2 on the activation of the T cell signaling cascade. Therefore, isolated T cells were stimulated by cross-linking with anti-CD3 antibodies for 3, 10 and 60 minutes. Analyses of the TCR-induced signaling cascade revealed a reduced phosphorylation of PLCγ (**Figures 5A, B**). PLCγ is an essential effector in the TCR signaling cascade and is capable to regulate various signaling pathways due to the hydrolysis of membrane lipid phosphatidylinositol 4,5-bisphosphate (PIP_2_) and the resulting generation of diacylglycerol (DAG). DAG on the other hand induces NF-κB and the RAS-Erk pathway (37, 38). In accordance with this, activation of the MAP-Kinase Erk down-stream of PLCγ was also reduced in T cells harboring the SHP2-D61Y mutation. Signaling proteins upstream of PLCγ like ZAP70 and LAT were not affected. In contrast to the altered MAP-Kinase Erk, there was no change in the activation of the MAP-Kinase JNK which depends on other signaling pathways (**Figures 5A, B**).

### pTα^SHP2mut^ mice control Influenza A Virus infection and produce higher frequencies of Tem CD8^+^ T cells

**Figure 6:**
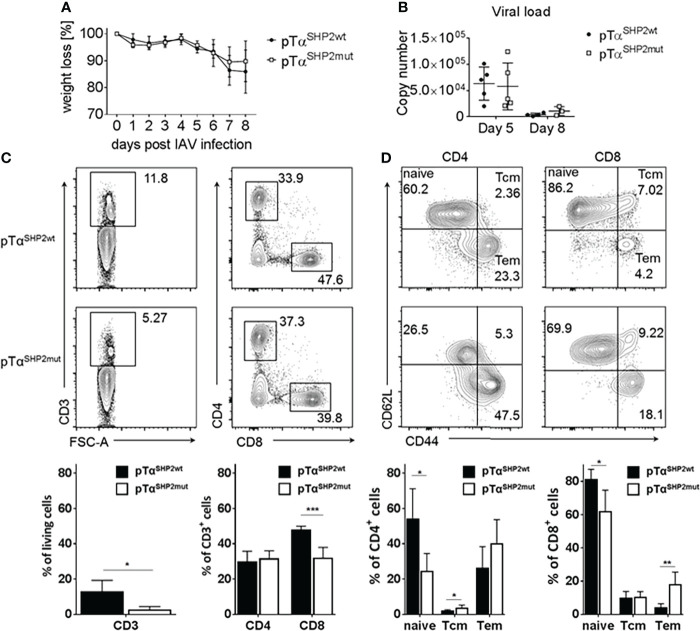
Comparable viral clearing and higher Tem frequencies in pTα^SHP2mut^ mice. Mice were infected for 8 days with PR8-OT-I IAV. **(A)** Body weight was monitored throughout the course of infection (5 mice per group). **(B)** Mice were sacrificed on day 5 and day 8 to monitor viral load in the lung, representative for 5 mice per group. **(C, D)** Cells were isolated from bone marrow and gated on single living CD3^+^, CD8^+^, CD4^+^ and CD44^high^CD62L^low^ Tem as well as CD44^high^CD62L^high^ Tcm. Data is representative for 2 independent experiments with n=4 mice per group. (*p<0.05, **p<0.01, ***p<0.001, Student’s t-test, mean + SD).

To analyze whether the SHP2-D61Y mutation in CD8^+^ T cells influences memory formation and whether it provides enhanced protection against viral infection, mice were challenged with the recombinant IAV strain PR8-OT-I (H1N1) harboring the MHC class I ovalbumin SIINFEKL-epitope. The pTα^SHP2mut^ and Cre-control mice displayed comparable weight loss during the course of IAV infection (**Figure 6A**). In accordance with those findings, viral load in the lung was similar between both experimental groups at the peak of disease (at 5 d.p.i.) and was almost cleared 8 days post infection, indicating that T cells of SHP2-D61Y mutant mice are able to inhibit viral replication and to promote viral clearance to the same extent as control mice (**Figure 6B**).

After virus elimination, the bone marrow is one of the preferential homing sites where long-lived memory T cells can reside and undergo homeostatic proliferation until re-encounter with a pathogen (39–41). To analyze the T cell composition within the bone marrow T cell subsets were determined at 8 days following IAV infection. Although higher frequencies of T cells with a memory phenotype were observed in the bone marrow, frequencies of CD3^+^ T cells were strongly reduced in pTα^SHP2mut^ mice after IAV infection (**Figure 6C**) which is similar compared to the steady state analysis of mice (**Figure 2A**). In addition, a significant reduction of CD8^+^ T cells could be observed within the bone marrow of pTα^SHP2mut^ mice (**Figure 6C**). In both, CD4^+^ and CD8^+^ T cell subsets, fewer frequencies of naïve T cells were observed accompanied by a shift to significant higher frequencies of Tem at day 8 following IAV infection in the CD8**^+^** T cell subset of mice carrying the constitutive active SHP2 mutation (**Figure 6D**). Thus, these data point again to a role of the SHP2-D61Y mutation in T cells to enhanced formation of a memory T cell phenotype which is even increased upon infection.

### Increased homing of IAV-specific CD8^+^ Tem into the bone marrow of pTα^SHP2mut^ mice

The observation that CD8^+^ T cells were reduced in the bone marrow of pTα^SHP2mut^ mice, but Tem frequency was increased upon IAV infection, raised the question whether IAV infection leads to an increase of virus-specific T cells in this organ. The virus used in this study contains the SIINFEKL epitope sequence in the neuraminidase gene, which is generated in IAV infected cells together with viral proteins. Subsequently, the SIINFEKL-epitope is presented *via* H-2Kb/MHC class I molecules and recognized by epitope-specific CD8^+^ T lymphocytes, thus allowing the quantification of IAV-specific CD8^+^ Tem by pentamer-staining. Only a few IAV-specific CD8^+^ T cells were observed in the lungs of both pTα^SHP2mut^ and Cre-control mice at day eight post infection indicating that the lung-resident virus-specific T cell pool had already contracted at the time the viral infection was cleared (**Supplementary Figure 4**, top). In contrast, significant higher frequencies of virus-specific CD8^+^ Tem cells could be found within the bone marrow of pTα^SHP2mut^ mice (**Supplementary Figure 4**, bottom).

### Reduced IFNγ-production by virus-experienced pTα^SHP2mut^ T cells upon *ex vivo* re-stimulation

**Figure 7:**
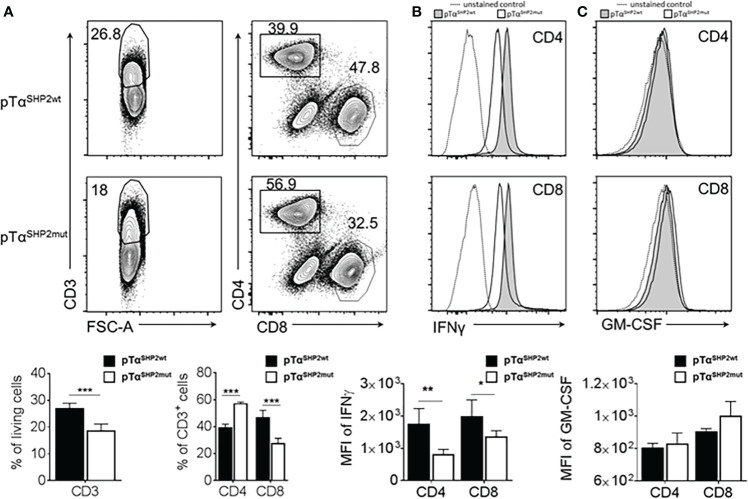
Altered T cell composition and reduced IFNγ production in pTα^SHP2mut^ mice. Splenocytes of PR8-OT-I IAV infected (8 days) SHP2 mutant and control mice were stimulated with 5µg/mL plate-bound α-CD3 and 1µg/mL soluble α-CD28 for 16 h. **(A)** FACS plots and graphs show frequencies of CD3^+^ T cells within living cells and shifted CD4^+^:CD8^+^ T cell ratio within the CD3^+^ T cell population. **(B)** Reduced levels of IFNγ in pTα^SHP2mut^ cells were observed within the CD4^+^ and CD8^+^ T cell subset and **(C)** similar levels of GM-CSF expressed in both CD4^+^ and CD8^+^ T cell subsets. Histograms show the representative mean fluorescence intensities of fluorochrome-conjugated antibody against IFNγ and GM-CSF for pTα^SHP2wt^ mice (grey), pTα^SHP2mut^ mice (white) and unstained control (MFI, dashed). Individual value plot represents the mean of fluorescence intensity. Data is representative for 2 independent experiments with n=3 SHP2 mutant or 5 control mice. (*p<0.05, **p<0.01, ***p<0.001, Student’s t-test, mean + SD).

To analyze the impact of the SHP2-D61Y mutation on T cell effector function, splenocytes of IAV-infected mice were re-stimulated at day 8 post infection with anti-CD3/CD28. As observed before, the T cell composition of stimulated cells was altered in SHP2-D61Y mutant T cells towards lower frequencies of CD3^+^ T cells and a shift in the CD4:CD8 T cell ratio to lower frequencies of CD8^+^ CTLs and higher numbers of CD4^+^ T cells (**Figure 7A**). Further analysis of intracellular IFNγ-synthesis revealed reduced levels of this pro-inflammatory cytokine in both CD4^+^ as well as CD8^+^ T cell subsets (**Figure 7B**). Besides IFNγ, GM-CSF is one of the major cytokines released by T cells orchestrating the crosstalk between adaptive and innate immune responses during inflammation and infection (42). Moreover, it has been shown that high GM-CSF concentrations protect the host from lethal IAV-infection (43). Therefore, GM-CSF levels produced by *ex vivo* stimulated CD4^+^ and CD8^+^ T cells were measured. Although a tendency to a higher amount in the CD8^+^ T cell compartment could be observed, differences were not significant (**Figure 7C**).

In summary, viral infection can be controlled by T cells with the SHP2-D61Y mutation to the same extent as by wild-type T cells. As a result of IAV-infection, mice harboring the mutation produce increased numbers of memory T cells (especially CD8^+^ Tem cells) that are recruited to the bone marrow. However, T cells with the SHP2-D61Y mutation exhibit lower effector functions compared to Cre-control T cells as measured by reduced IFNγ-production.

## Discussion

In this study we demonstrated that the SHP2-D61Y mutation exclusively expressed in T cells did not result in the onset of a proliferative disorder, whereas *Ptpn11^D61Y^* expression in mice with the Cre recombinase driven by the *cd4* promoter led to splenomegaly probably caused by an increase of total cell numbers in the spleen (**Figure 1**). Previously published data indicate that the SHP2-D61Y mutation expressed in all hematopoietic cells renders myeloid progenitors to be more susceptible towards proliferation due to cell-autonomous and cytokine-mediated (GM-CSF or M-CSF) effects (44). When we analyzed expression levels of the growth factor GM-CSF in T cells following IAV infection, *Ptpn11^D61Y^* T cells did not display significantly increased GM-CSF levels, which therefore do not necessarily indicate a positive feedback loop to enhance cell proliferation as observed in the mice described by Chan et al. (26). Remarkably, in that study the relative number of splenic T cells was decreased in mice carrying the SHP2-D61Y mutation in hematopoietic cells, probably due to a compensatory effect (26). In contrast, pan-hematopoietic cell expression of the E76K mutant led to acute myeloid leukemia as well as T and B cell leukemia indicating that the particular phenotype depends on the *Ptpn11* mutation and the site of expression (25).

### T cell subset distribution and memory formation in pTα^SHP2mut^ mice

Divergent data regarding the role of SHP2 in T cells have been published. While on the one hand SHP2 was shown to be indispensable for T cell development and signaling (12), on the other hand it turned out to be dispensable for development and generation of virus-specific T lymphocytes (24).

In our model thymic selection seemed not to be altered in T cells carrying the SHP2-D61Y mutation, although reduced frequencies of CD3^+^ T cells in the bone marrow of young (10 to 14 weeks) pTα^SHP2mut^ mice could be observed. In the aged (11-14 months-old) cohort and in IAV-infected pTα^SHP2mut^ mice, this effect was even more pronounced and was also visible in the spleen. Coincidently, the number of CD4^+^ T cells was increased and lower frequencies of CD8^+^ T cells could be detected in these organs (**Figures 2**, **3**, **6**, **7** and **Supplementary Figure 3**). The decrease of CD8^+^ T cells could be explained by the fact that T cells expressing the mutant *Ptpn11^D61Y^* underwent cell death (late apoptosis/necrosis) significantly more often than control T cells (**Figure 4**) and is supported by previously published data displaying that overexpression of wild-type SHP2 in T cells leads to a significant increase of apoptosis (45). SHP2 is further known to inhibit TCR signaling on two levels, either due to direct de-phosphorylation of signaling proteins such as ZAP70 or due to the inhibition of signaling *via* PD-1 (17, 19). So far, it is not clear to which extent these pathways are affected by the SHP2-D61Y mutation, this could be the topic of a follow up study. Here, it would be possible to use T cells from pTα^SHP2mut^ together with a PD-1 inhibitor to dissect the contribution of SHP2 to either PD-1 or TCR signaling. However, the reduced proliferation observed by stimulation with low CD3 antibody concentrations **(** **Figure 4**) indicates that the T cell activation threshold of SHP2-D61Y-mutant T cells is elevated. As a consequence, frequent interactions between TCRs and peptide/MHC complexes especially in the periphery (e.g. in the spleen) might not be sufficient to induce tonic-TCR signaling required for survival and homeostatic proliferation of naïve T cells (46, 47). In addition, altered frequencies of CD4^+^ and CD8^+^ T cells in the spleen and bone marrow could be the result of a modified recruitment of gain-of-function mutation expressing cells to these organs.

Despite reduced numbers of CD8^+^ T cells in the spleen and bone marrow of young, aged and IAV-infected mice, significant enhanced levels of cell adhesion molecule CD44 expressing effector memory T (Tem) cells, most of them being CD8 positive, could be observed under these conditions. On T cells, surface receptor CD44 mediates adhesion, rolling and homing of lymphocytes from the blood to lymphoid organs or inflamed tissues (48). CD44 is upregulated after antigen recognition and activation of naïve T lymphocytes and remains elevated on memory T cells which protect the host against re-infection (49). In addition, CD44 expression is required for T cell survival during and after clonal expansion where increased CD44 can prevent activation-induced cell death (AICD) (49–51). It has been shown that CD44 can directly interact with Lck and induces a TCR-like signaling cascade, thereby circumventing PD-1 induced down-regulation of TCR signaling pathways (52). Based on this, only T cells expressing higher levels of CD44 might persist as in particular observed in aged pTα^SHP2mut^ mice. In contrast, CD44 has also been shown to promote apoptosis under some conditions indicating that CD44-related functions can vary depending on the (disease) model studied (53). In analyses using the PTP inactive cysteine^459^ to serine mutation, a similar up-regulation of CD44 in T cells was observed, indicating that this process is not caused by the PTPase activity of SHP2 but might rather be the result of its adapter functions, such as forming a complex with growth factor receptor-bound protein 2 (GRB2), and the induction of down-stream signaling pathways (22, 54).

The phosphatase SHP1, which strongly interferes with the TCR signaling cascade by altering T cell effector functions such as the production of CD8^+^ effector cells, has no impact on the formation of memory T cells (55). This might support the hypothesis that an active SHP2 is required for differentiation into memory T cells. Due to the possible redundancy of the two phosphatases SHP1 and SHP2 only a deficiency of both phosphatases in T cells might further unravel their possible functions.

Effector memory CD8^+^ T cells have previously been characterized by their cytolytic function and localization in the blood and peripheral sites such as inflamed tissue (56, 57). Data obtained from analysis of human T cells regarding differentiation, homeostasis and persistence revealed that CD8^+^ Tem clones are broadly distributed among blood, lymphoid and mucosal sites and further undergo age-associated changes (58). Our analysis revealed significant higher frequencies of virus-specific effector memory CD8^+^ T cells which could be retraced within the bone marrow of pTα^SHP2mut^ mice after IAV-infection (**Supplementary Figure 4**). In comparison, previously published data indicate that SHP2-deficiency in T cells does not affect the formation of virus-specific memory CD8^+^ T cells. However, it should be noted that a different mouse model was used for these studies (24).

### Signaling and proliferation of *Ptpn11^D61Y^* expressing T cells

Analyzing proteins of the TCR signaling cascade revealed a reduced phosphorylation of PLCγ in *Ptpn11^D61Y^* T cells (**Figure 5**). PLCγ in T cells plays an important role not only in TCR-mediated proliferation but also in AICD as mice which are deficient for PLCγ in T cells exhibited more activation-induced cell death of peripheral T cells. This observation was accompanied by an activated T cell phenotype probably induced by lymphopenia and a reduced number of regulatory T cells (59). Although T cells in our model display a similarly activated phenotype, mice carrying the SHP2 gain-of-function mutation did not develop autoimmune phenomena as observed by Fu and colleagues. We found reduced phosphorylation and activation of MAP-Kinase Erk in SHP2-D61Y mutant T cells as well as decreased T cell proliferation and diminished IFNγ-synthesis of IAV-experienced T cells upon *ex vivo* TCR stimulation (**Figures 4**, **5**, **7**). These results are also supported by the data obtained from mice with T cell-specific deletion of PLCγ displaying impaired TCR-induced proliferation, Erk-phosphorylation and activation of the T cell transcription factors AP-1, NFAT, and NF-κB. Reduction in transcription factor activation subsequently leads to a reduced cytokine production which is crucial for T cell proliferation and might further explain the observed reduction of proliferation *in vitro* upon antibody stimulation (59). However, our data indicate that CD25 upregulation is comparable between pTα^SHP2wt^ and pTα^SHP2mut^ mice (data not shown) which suggests that at least IL-2 production is not affected by the SHP2-D61Y mutation.

Furthermore, SHP2 is proposed to influence T cell signaling through its incorporation into the LAT complex *via* binding of GRB2 (60, 61). This might serve as an explanation for the reduced phosphorylation of PLCγ, which is also recruited to the LAT-complex, and which might be prevented due to interference with the SHP2-GRB2-LAT-complex (62). The observed reduced phosphorylation of PLCγ results in a subsequent reduction of Erk-phosphorylation which is downstream of PLCγ, whereas the phosphorylation of proteins upstream of the LAT complex, such as ZAP70, or the phosphorylation of LAT itself, remains unaffected (**Figure 5**).

Thus, only subliminal triggering of the TCR, reflecting the signals required for survival and homeostatic proliferation in the periphery, appeared to be affected in pTα^SHP2mut^ mice. Strong stimuli, such as high antibody concentrations or co-stimulation (data not shown) had no effect on the T cell signaling cascade and subsequent proliferation *in vitro*. Similarly, the course of influenza infection induced a robust T cell response and was not affected in pTα^SHP2mut^ mice. These results indicate that SHP2 has a mild effect on the activation threshold in T cells and further support the proposed role of SHP2 as being responsible for fine-tuning T cell signaling processes (63).

Analyzing the role of the gain-of-function mutation SHP2-D61Y in T cells, we used a mouse model expressing the SHP2 mutant in conditional mice with the Cre recombinase driven by the *Ptcra*-promoter. In summary, our data support the role of SHP2 in T cell signaling (60). In addition, permanently enhanced SHP2 phosphatase activity in T cells also exerts effects on proliferation and survival as well as on the formation of memory T cells. However, since SHP2-function in T cells is multilayered and varies under different conditions, the corresponding SHP2-induced phenotype cannot be predicted and therefore must be assessed separately. In conclusion, our data add information to the function, associated signal cascades and side effects regarding the mode of action of immunomodulatory compounds such as PD-1 checkpoint inhibitors.

## Data availability statement

The raw data supporting the conclusions of this article will be made available by the authors, without undue reservation.

## Ethics statement

The animal study was reviewed and approved by Landesverwaltungsamt Sachsen-Anhalt.

## Author contributions

CC, NI, DB and US designed and organized the experiments. CC, NI, SF, AJ and ET conducted the experiments. CC, NI and AJ analysed data. CC, NI, SF, AJ, TS, LS, MZ, HF, BS, DB and US interpreted data. CC, NI and US wrote the paper with input from TS, LS and DB. DB and US supervised the study. All authors contributed to the article and approved the submitted version.

## Funding

This work was supported by the European Regional Development Fund (EFRE, 2014 – 2020; project number GHS-20-0031) to US and the Helmholtz Initialization and Networking Fund for Infection Research Greifswald (ZoonFlu; 2021) to DB and US. We acknowledge support by the DFG (Deutsche Forschungsgemeinschaft) within the CRC854 (Project-ID 97850925-SFB 854) to TS, LS, BS, DB and US. MZ was supported by grants from the German Federal Ministry of Education and Research - BMBF (German Network for RASopathy Research ‘‘GeNeRARe’’ [FKZ: 01GM1902A] and EJP-RD “NSEuroNet” [FKZ: 01GM1921A]). We acknowledge support for the Article Processing Charge by the German Research Foundation and the Open Access Publication Fund of the University of Greifswald.

## Acknowledgments

We thank Nadine Riemann, Xenia Gorny and Andrea Kröber for their initial support in the project. Conditional SHP2^D61Yflox^ (B6.129S6-Ptpn11tm1Toa/Mmjax) mice were kindly provided by B. G. Neel and T. Araki and Ptcra^tm1(icre)Hjf^ mice by H. J. Fehling.

## Conflict of interest

The authors declare that the research was conducted in the absence of any commercial or financial relationships that could be construed as a potential conflict of interest.

## Publisher’s note

All claims expressed in this article are solely those of the authors and do not necessarily represent those of their affiliated organizations, or those of the publisher, the editors and the reviewers. Any product that may be evaluated in this article, or claim that may be made by its manufacturer, is not guaranteed or endorsed by the publisher.
