# Supplementary material for: T cell-specific constitutive active SHP2 enhances T cell memory formation and reduces T cell activation
Source: Front Immunol. 2022 Aug 2;13:958616. doi: 10.3389/fimmu.2022.958616 (PMC9379337; doi:10.3389/fimmu.2022.958616)
Supplement: Supplementary file 1 [file DataSheet_1.docx]

Supplementary Material


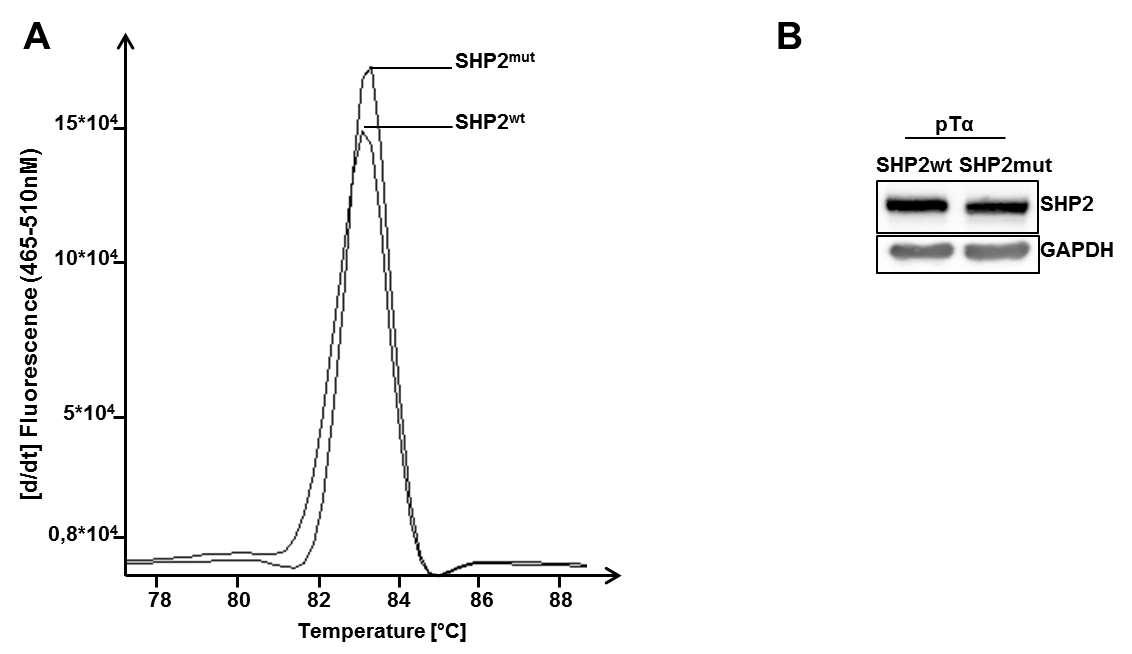


**Supplementary Figure 1**. Identification of SHP2-D61Y mice

Genomic DNA of wildtype (SHP2wt) and mutant (SHP2mut) mice were analyzed following qPCR with the LightCycler480 instrument by high resolution melting (HRM) method (A). SHP2 protein-expression in purified T cells of pTα^SHP2wt^ and pTα^SHP2mut^ mice (B). GAPDH served as loading control.


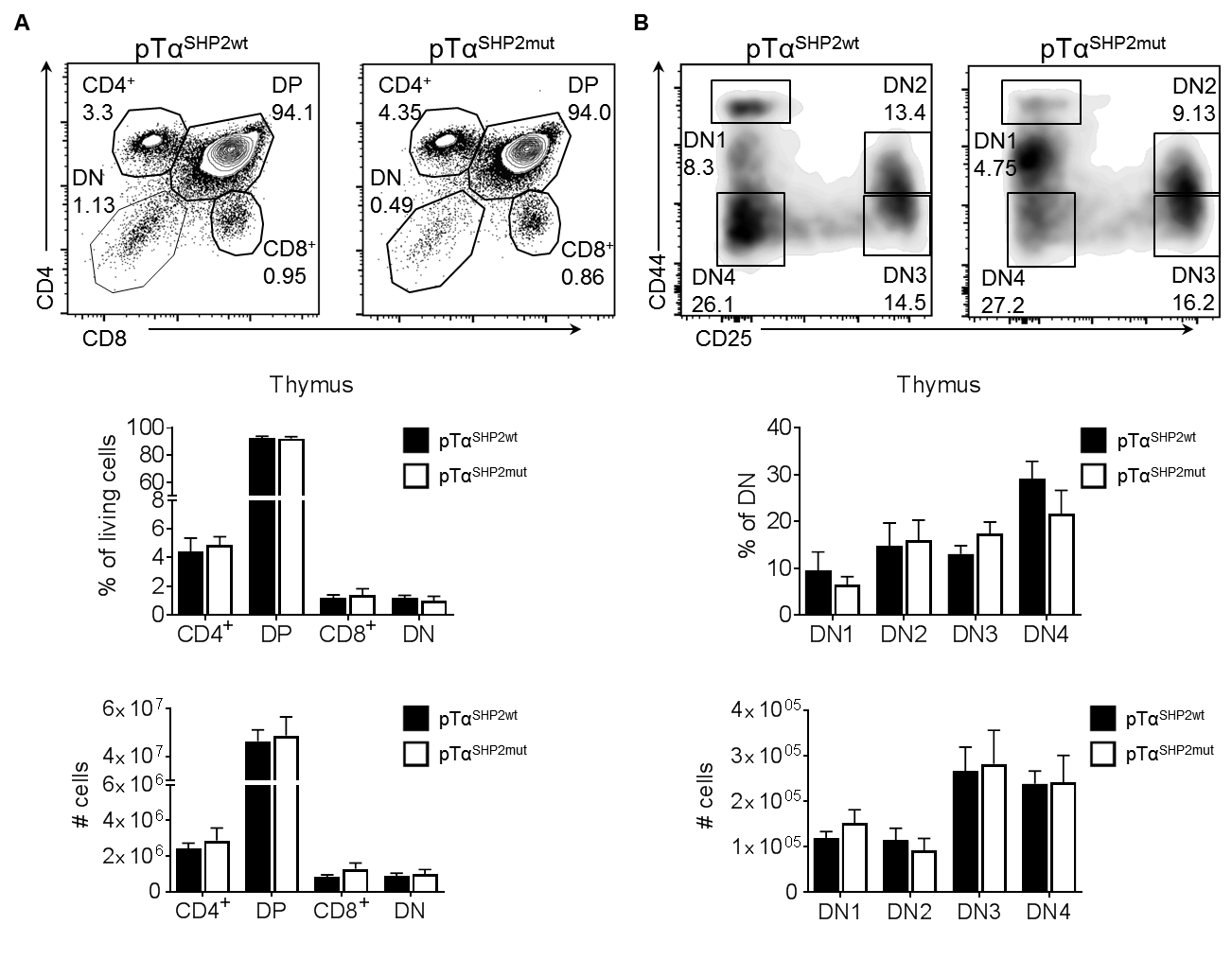


**Supplementary Figure 2.** No alteration in thymic T cell development in mice carrying the SHP2- D61Y-mutation

Cells isolated from the thymus of naïve pTα^SHP2mut^ mice and pTα^SHP2wt^ control mice were gated on single, living cells and used for further FACS analysis. (A) FACS plots and graph show frequencies of double positive, double negative and CD4^+^ or CD8^+^ single positive T cells (upper panel) and cell numbers calculated on counted living cells (bottom). (B) Double negative cells were further subdivided into 4 DN stages according to the expression of CD44 and CD25. FACS plots and graph show frequencies of DN1, DN2, DN3 and DN4 cells (upper panel) and cell numbers calculated on counted living cells (bottom). Data are representative for 2 independent experiments with n=5 mice per group.


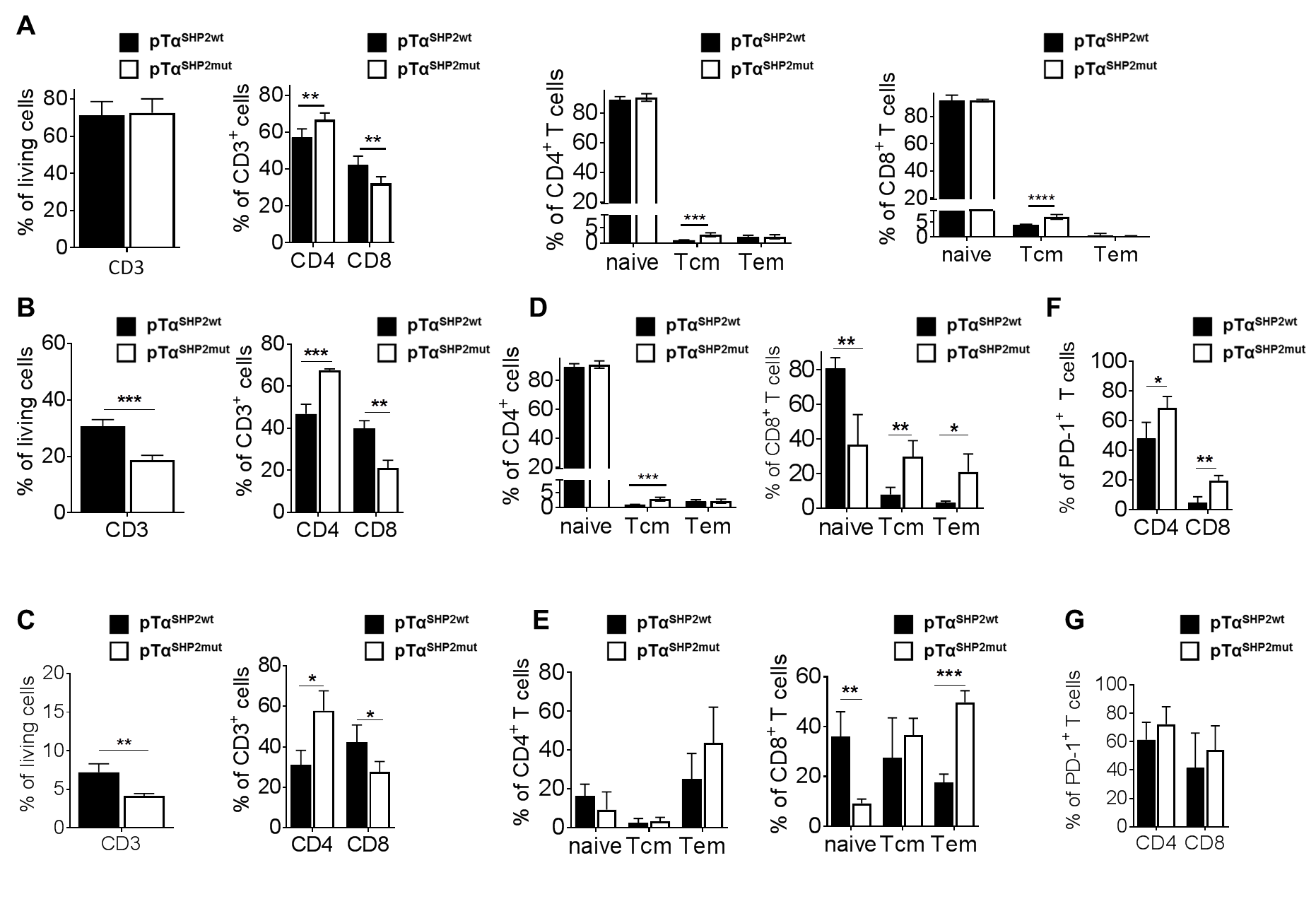


**Supplementary Figure 3.** Reduced T cells and shift in the naïve:Tem T cell ratio in aged mice

Cells isolated from lymph nodes of naïve pTα^SHP2mut^ and Cre-control mice were gated on single living cells and used for further FACS analysis. (A) Graphs show frequencies of CD3^+^ T cells within the lymph nodes, the CD4:CD8 T cell ratio and proportion of naïve, central memory and effector memory T cells in both CD4^+^ and CD8^+^ T cell subsets in pTα^SHP2wt^ and pTα^SHP2mut^ mice.

Data is representative for 2 independent experiments with n=5 mice. (**p<0.01, ***p<0.001, ****p<0.0001, Student‘s t-test, mean + SD).

Cells isolated from spleen and bone marrow of 11-14 month-old naïve pTα^SHP2mut^ and pTα^SHP2wt^ control mice were gated on single living cells and used for further FACS analysis. Graphs show reduced frequencies of CD3^+^ T cells and a shift to lower frequencies of CD8^+^ T cells in (B) spleen and (C) bone marrow. FACS plots and graphs show proportion of naïve, central memory and effector memory T cells in both CD4^+^ and CD8^+^ T cell subsets within the spleen (D) and the bone marrow (E). Frequencies of PD1 expressing CD3^+^ T cells were analyzed in spleen (F) and in bone marrow (G). Data is representative for 2 independent experiments with n = 3 control and 4 pTα^SHP2mut^ mice. (*p<0.05, **p<0.01, ***p<0.001, Student‘s t-test, mean + SD).


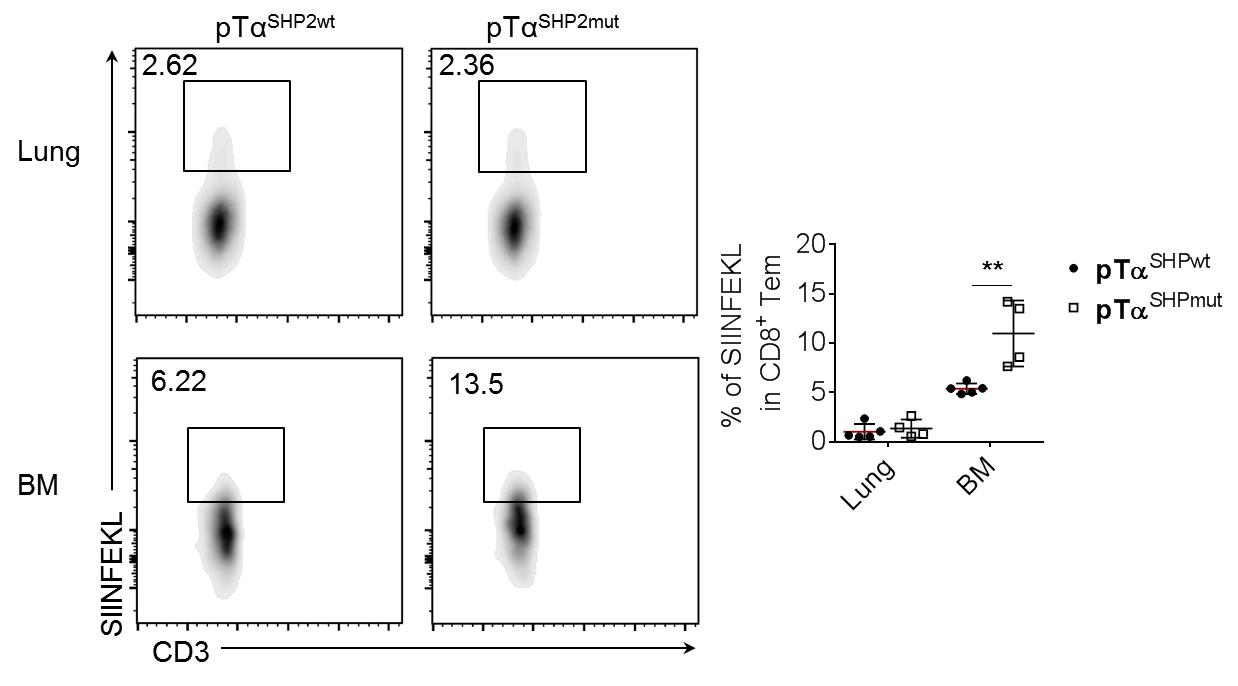


**Supplementary Figure 4.** Enhanced IAV-specific T cells are located in the bone marrow of SHP2 mutant mice

Mice, intranasally infected with PR8-OT-I IAV, were sacrificed at day 8 post infection. Cells were isolated from lungs and bone marrow and gated on single living CD3^+^, CD8^+^ and CD44^high^CD62L^low^ Tem. IAV-specific T cells were identified using fluorochrome-labeled pentamer specific antibody for the OVA peptide SIINFEKL. Graph shows frequencies of IAV-specific Tem in lung and bone marrow (BM). Data is representative for 3 independent experiments with n = 5 control and 4 SHP2mut mice. (**p<0.01, Student‘s t-test, mean + SD).
